# Supplementary material for: Assembling of Carbon Fibre/PEEK Composites: Comparison of Ultrasonic, Induction, and Transmission Laser Welding
Source: Materials (Basel). 2022 Sep 13;15(18):6365. doi: 10.3390/ma15186365 (PMC9503134; doi:10.3390/ma15186365)

Supplementary Materials

Figure S1. FTIR spectra.

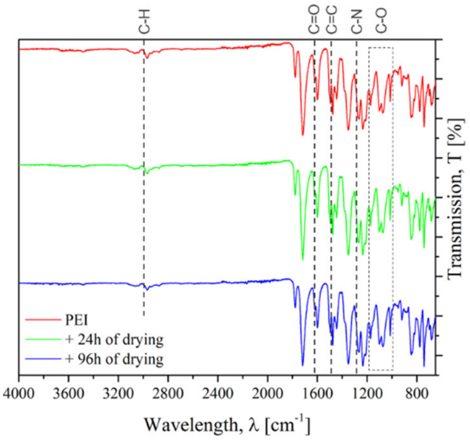

(a) Effect of drying on the chemical structure of the PEI

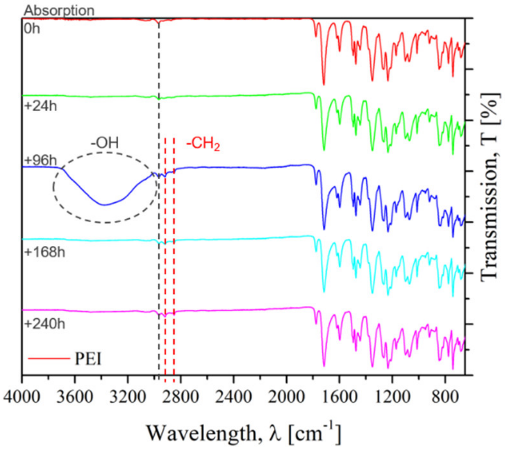

(b) Effect of absorption for PEI

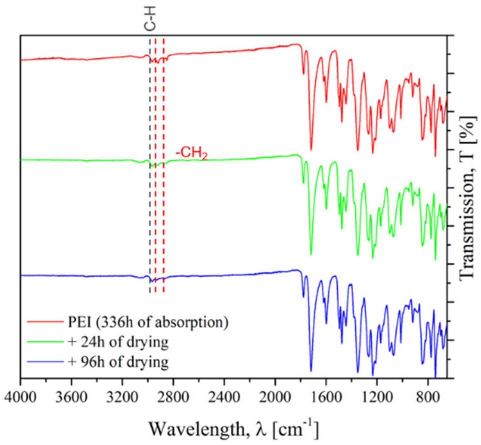

(c) Effect of desorption for PEI

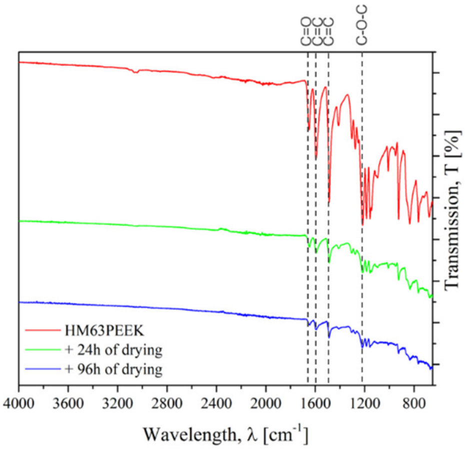

(d) Effect of drying on the chemical structure of the HM36/PEEK

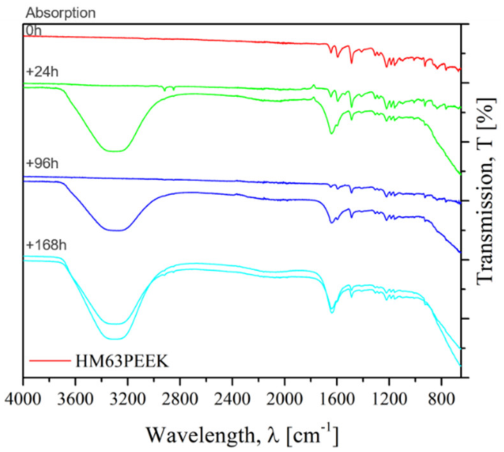

(e) Effect of absorption for HM63/PEEK

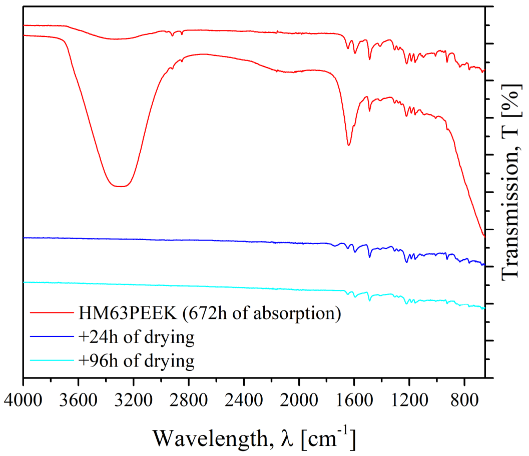

(f) Effect of desorption for HM63/PEEK

Figure S2. Absorbance of PEI with sample thickness (dots) - Beer-Lambert law (dotted red line).

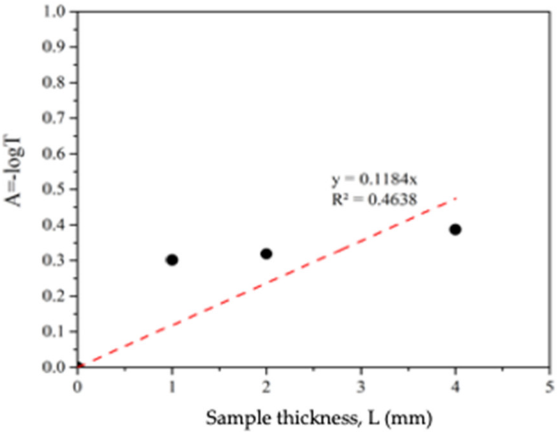

Supplement: Supplementary file 1 [file materials-15-06365-s001.zip › materials-1884876-supplementary.pdf]
